# Ripples across generations: offspring outcomes after parental infant institutionalisation—study protocol of the LifeStories Offspring Project

**DOI:** 10.3389/fpsyg.2026.1736639

**Published:** 2026-07-02

**Authors:** Patricia Lannen, Nina Graf, Selin Kilic, Raquel Paz Castro, Flavia M. Wehrle, Oskar G. Jenni

**Affiliations:** 1 Marie Meierhofer Children’s Institute, University of Zurich, Zurich, Switzerland; 2 University of Zurich, Zurich, Switzerland; 3 University of Basel, Basel, Switzerland; 4 Child Development Center, University Children’s Hospital Zurich, Zurich, Switzerland; 5 Children’s Research Center, University Children’s Hospital Zurich, Zurich, Switzerland

**Keywords:** infant institutionalization, intergenerational transmission, life-course studies, psychosocial deprivation in early childhood, social and compulsory measures in Switzerland

## Abstract

Intergenerational transmission of adversity is increasingly studied, yet little is known about the transmission of specific early-life experiences, particularly deprivation-related experiences. The LifeStories Offspring Project addresses this gap by examining whether infant institutionalisation under conditions of psychosocial deprivation is associated with outcomes in the next generation. The study builds on a population-based, non-selective cohort of individuals placed in Swiss infant care institutions between 1958 and 1961 under social and compulsory measures before the 1981 law reform. The parent generation was first assessed in infancy, followed up in adolescence, and reassessed approximately 60 years later. Compared with a non-institutionalised community cohort from the Zurich Longitudinal Studies, formerly placed individuals showed poorer physical and mental health, poorer cognitive functioning, and increased mortality. The current study will examine the adolescent and adult offspring of formerly institutionalised individuals (*N* = 163) and compare them with offspring from the non-affected ZLS cohort. Using questionnaires, neuropsychological assessments, and semi-narrative interviews, the study will investigate physical, cognitive, socioemotional, and socioeconomic outcomes, as well as potential pathways of transmission. Quantitative data will be analysed using longitudinal statistical techniques, including latent growth models, latent mediation and moderation models, and random-intercept cross-lagged models. By focusing on adult “children” of individuals exposed to a clearly defined context of early psychosocial deprivation, this globally unique study moves beyond general adversity frameworks and threat-based models of intergenerational transmission. It investigates how the absence of early caregiving experiences may reverberate when those affected become parents themselves, while also contributing to societal recognition and reconciliation related to compulsory social measures in Switzerland.

## 1 Introduction

Mounting evidence demonstrates that experiences that profoundly shape an individual’s life may not only affect the individuals themselves (Felitti et al., 1998; Brown et al., 2009; Hughes et al., 2017), but may also be associated with outcomes in the next generation, including physical, cognitive, and socioemotional/behavioral outcomes. Such intergenerational associations have been most prominently documented in survivors of war trauma and other severe stress- or threat-related experiences (Scheering and Zeanah, 2001; Bowers and Yehuda, 2016). Proposed mechanisms include biological and epigenetic pathways, such as neuroendocrine and stress-related processes (Bowers and Yehuda, 2016), as well as psychosocial and behavioral pathways, including parental sensitivity, emotion regulation, attachment-related functioning, and parenting behavior. These mechanisms are relevant because parental sensitivity and emotion regulation are closely related to early attachment experiences (Enlow et al., 2014; Pat-Horenczyk et al., 2015; Deans, 2020), and parental attachment style itself has been shown to correlate with offspring attachment style (van IJzendoorn, 1995; Magai et al., 2000; Steele et al., 2003).

Further pathways of intergenerational transmission may operate through socioeconomic, cognitive, mental health, and health-behavioral mechanisms. Studies indicate that parental socioeconomic status (SES) is related to a range of offspring outcomes, including cognitive abilities (Delis et al., 2001; Osler et al., 2013; Zhang, 2020), health (Gilman et al., 2002; Luo and Waite, 2005; Galobardes et al., 2006; Power et al., 2007), and offspring SES (Sirniö et al., 2013; Karagiannaki, 2017; Vauhkonen et al., 2017). Moreover, cognitive abilities of parents correlate with cognitive abilities of offspring (Black et al., 2009; Anger, 2012), parental mental health is associated with mental health in offspring (McLaughlin et al., 2012; Vandeleur et al., 2012; Jaffee et al., 2021), and parental health behavior has been documented to affect offspring health (Frank et al., 2001; Sokol et al., 2003; Schuetze et al., 2006). At the same time, risk and protective factors, such as the availability of a caring adult, a sense of self-efficacy, or optimism as a personality trait, may shape resilience processes and contribute to differential developmental trajectories (Masten and Barnes, 2018).

However, there is increasing evidence that adverse experiences need to be considered in a more differentiated way, rather than relying on sum scores that combine different types, frequencies, and intensities of adversity (McLaughlin and Sheridan, 2016; McLaughlin et al., 2019). In particular, theoretical models distinguish between threat-related experiences, such as violence, abuse, or exposure to danger, and deprivation-related experiences, which are characterized by the absence of expected environmental inputs and developmentally necessary stimulation (McLaughlin and Sheridan, 2016; McLaughlin et al., 2019). This distinction is also important for the study of intergenerational transmission. While much of the existing literature has focused on stress, trauma, and maltreatment, including biological stress-related pathways and social learning processes in the transmission of aggressive or abusive parenting behavior (Bowers and Yehuda, 2016), considerably less is known about whether and how deprivation-related experiences (neglect) are associated with outcomes in the next generation.

Within this framework, evidence on neglect remains particularly scarce, a gap that has been described as the “neglect of neglect” (Stoltenborgh et al., 2013). Moreover, studies on neglect often do not distinguish between physical neglect and psychosocial neglect, also referred to as psychosocial deprivation. Physical neglect refers primarily to the absence of adequate nutrition, hygiene, medical care, or physical safety, whereas psychosocial deprivation refers to the absence or severe insufficiency of developmentally necessary emotional, social, and cognitive stimulation. Psychosocial deprivation may include limited sensitive and contingent caregiver–child interaction, little comforting or emotional co-regulation, few opportunities for reciprocal social engagement, scarce language input, and restricted access to age-appropriate play, exploration, and learning experiences. In many contexts, psychosocial deprivation co-occurs with physical neglect, including low hygiene standards, insufficient medical care, and inadequate nutrition, as documented, for example, in Romanian institutions after the fall of the Ceausescu regime (Groze and Ileana, 1996; Rutter, 1998).

The risk that psychosocial deprivation goes undetected is particularly high. Unlike physical neglect, it may not be immediately visible: children may appear adequately fed, clean, and physically safe, while still lacking the sustained relational, linguistic, and exploratory experiences that support socioemotional, cognitive, and neurodevelopmental processes. Its consequences may therefore emerge only gradually, for example in delays in language, self-regulation, social reciprocity, attachment-related behaviors, or cognitive development, rather than as acute signs of harm. In addition, the extent of psychosocial deprivation is often difficult to document retrospectively, because routine records typically capture physical care, medical events, or placement histories more readily than the quality, frequency, and contingency of everyday caregiver–child interactions (Maclean, 2003; Zeanah and King, 2022). Thus, even when children’s immediate physical safety is not endangered, psychosocial deprivation poses a serious developmental risk (van IJzendoorn et al., 2020; Sand et al., 2024a, b).

With regard to the next generation, no study has been identified that specifically examines intergenerational associations of early psychosocial deprivation. This is an important gap because psychosocial deprivation is characterized by the absence of experiences, such as nurturing care, contingent responding, emotional co-regulation, and reciprocal interaction, that may later be particularly relevant when individuals become parents themselves. In contrast to threat-related models, in which transmission may involve the reproduction of abusive or aggressive behaviors through observation, imitation, reinforcement, internalized relational schemas, or parental representations (Egeland et al., 1988, 2002; Brown and Schormans, 2003; Pollak and Tolley-Schell, 2003), deprivation-related pathways may involve the long-term consequences of not having experienced reliable caregiving, emotional attunement, or developmentally supportive interaction. Potential mechanisms may therefore include altered emotion regulation, parental sensitivity, representations of caregiving relationships, expectations about children’s needs, and the quality of relational and communicative environments provided to the next generation.

In addition, evidence on intergenerational transmission into adolescence and adulthood remains scarce. Studying this specific form of early adversity is therefore crucial for understanding whether, and through which pathways, deprivation-related experiences may be associated with outcomes across generations. With a globally unique dataset and design, the present study will examine associations between parental infant institutionalisation under conditions of psychosocial deprivation and outcomes in adolescent and adult offspring, and will explore potential pathways of intergenerational transmission.

## 2 Methods and analysis

### 2.1 Design

The design is based on a quasi-experimental study that uses a population-based cohort of individuals placed in institutions as infants under psychosocial deprivation between 1958 and 1961. They were compared to a representative community sample cohort from the same time and geographic region of children growing up in families (see section 2.1.2.5). The 60-year longitudinal follow-up analysis of the cohorts revealed significant long-term effects of the institutional placement (Lannen et al., 2021, 2024, 2026; Sand et al., 2024b). The current study compares the offspring (adult “children”) of these two cohorts about 65 years after placement in infant care institutions of their parents.

**Figure 1:**
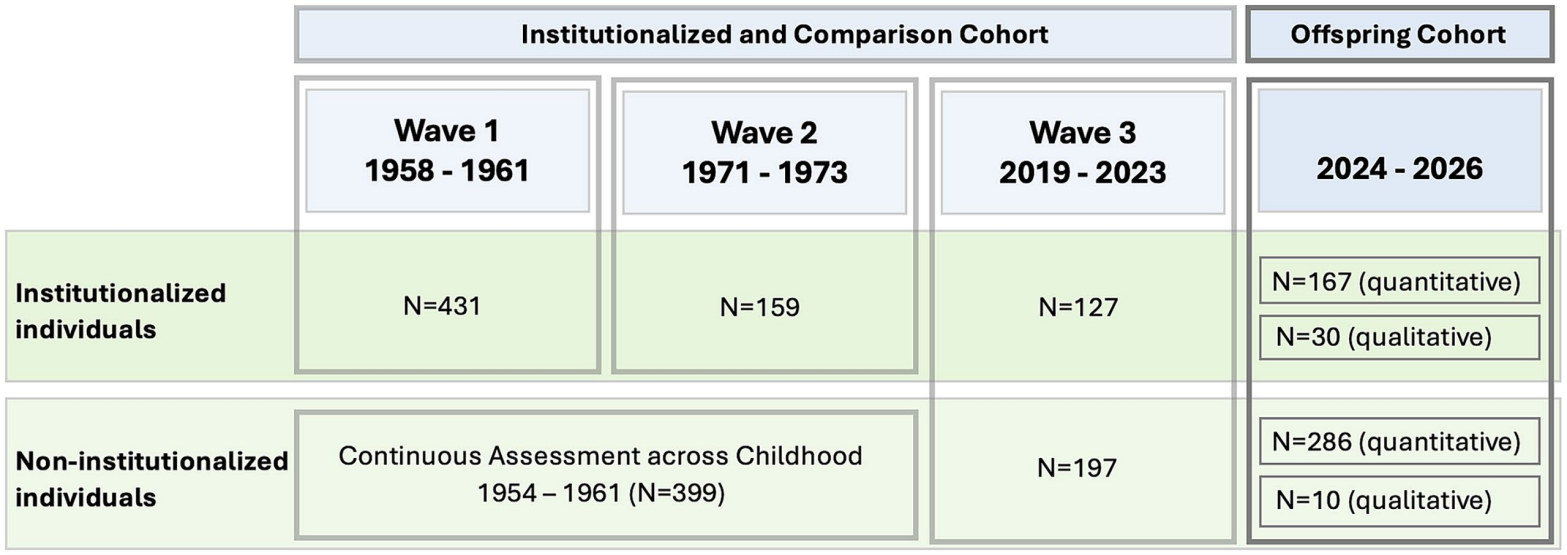
Study design of the formerly institutionalized and the comparison cohort and their offspring.

The proposed study uses a mixed method approach, retrospective and prospective information, combining historical and newly collected data from individuals placed in institutions as well as newly collected data from their offspring. The overall study design is depicted in Figure 1.

This article describes the cohort of offspring of those placed in institutions as infants. The comparison cohort not affected by institutionalization is described in detail in a separate study protocol (Wehrle et al., 2021).

#### 2.1.1 Assessment of formerly institutionalized individuals

The following subsections describe the parent generation of the present offspring study. They are included in the Methods and Analysis section because the historical placement context, the original population-based baseline assessment, and the subsequent follow-up assessments define the exposure, sampling frame, and longitudinal data structure on which the current intergenerational study is based.

##### 2.1.1.1 Historical and study context

In Switzerland, placing infants in institutions was quite common in the first half of the 20th century (Ryffel, 2013). The main reasons for having a child placed in an institution were either being an unmarried or under-aged mother or having a migrant status (Meierhofer and Keller, 1974). From the point of view of the authorities and society, having a child as a young, unmarried mother was slovenly (“liederlich”) and was to be “disciplined” (Ramsauer, 2000; see Lengwiler and Praz, 2018; Unabhängige Expertenkommission, 2019). Furthermore, migrant workers were subjected to serious prejudice and residence permit restrictions and were forced to work full time with long working hours in order to be able to remain in Switzerland. They therefore often had no choice but to place their children in infant care institutions (D’Amato, 2012; Joris, 2012).

Generally, infants were placed into institutions at a very young age, before the age of two weeks, due to the lack of paid maternity leave (Huber, 1995). Hard-earned success in reducing child mortality had made preventing the spread of germs a priority, so an institutional practice of “isolation” was the norm, involving as little physical contact as possible, feeding according to a rigid plan, and stringent hygiene (Ryffel, 2013).

Dr. Marie Meierhofer conducted a pioneering population-based study and collected developmental data on all 431 infants below age 3 years in 12 infant institutions in the canton of Zurich between 1958 and 1961 (Wave 1). The mean year of birth is 1957.4 (SD = 1.6). Some 51% of participants were female and 45% were of Swiss nationality. Due to migration status as a reason for placement, 55% of the cohort were of non-Swiss nationality, predominantly Italian. The data were compared to the data from the then available 1954–1961 cohort of the Zurich Longitudinal Studies (ZLS; N = 432) as a direct comparison with children from the same geographic location but raised within their families. Infants in institutions were exposed to serious psychosocial deprivation and experienced on average 55 min (*SD* 0.36 h) of interaction time in 24 h in the institutions (Sand et al., 2024a). Measured by Brunet-Lézine Scale (Brunet and Lézine, 1955), a state of the art standardized developmental test at the time, developmental delays in physical development, fine and gross motor skills, sociability and language skills for the institutionalized cohort compared to the non-institutionalized cohort were documented (Meierhofer and Keller, 1974; Sand et al., 2024a).

Dr. Meierhofer then conducted a hitherto unpublished follow-up study with 159 of these same children at the age of 14/15 years between 1971 and 1973 (Wave 2) and found clear evidence of possible negative long-term consequences of early psychosocial deprivation such as depression, stereotypical behavior, language and school-related problems, but also evidence of recovery (Meierhofer and Hüttenmoser, 1975).

##### 2.1.1.2 60-year follow-up

Between 2019 and 2023 a 60-year follow-up of the formerly institutionalized individuals as well as the comparison group took place. Details have been published in the study protocols (Lannen et al., 2021; Wehrle et al., 2021).

Through population registry search and in cooperation with the Federal Department of Foreign Affairs, 96% of formerly institutionalized individuals still residing in Switzerland and, 45% of individuals residing abroad were found (total N found = 358, 83%). Of those, 246 individuals were eligible to be contacted (ineligibility included death, contact ban, and suspicion of early adoption). In the end, 127 individuals (51.6%) of eligible participants took part in the assessment.

Importantly, participants of the 60-year follow-up did not differ from non-participants with regard to the developmental quotient at Wave 1 (B = −0.01, *p* = 0.29) or gender (B = 0.08, *p* = 0.75). However, individuals with an Italian nationality were less likely to complete the questionnaire as part of the 60-year follow-up than individuals with a Swiss nationality (B = −1.09, *p* = 0.02). This is likely due to the fact that descendants of migrant workers were more likely to have moved back to their country of origin and were therefore less likely to be successfully located 60 years later.

Participants completed a questionnaire on health and well-being, participated in a neuropsychological assessment (neuro-cognitive and neuro-motor skills) and biographical narrative interviews (Rosenthal, 2002).

The study found that the individuals formerly placed in institutions scored significantly worse on various outcomes: cognition (Sand et al., 2024b), physical and mental health (Lannen et al., 2024) as well as life satisfaction, emotion regulation, attachment and income (Lannen et al., 2024). Individuals were also about 1.5 times more likely to have already passed away if they were placed in institutions (Lannen et al., 2026). There is indication that the group differences are in fact related to the institutions because we found no differences in birth weight (*d* = −0.14, *p* = 0.19) and the placement happened right after birth, suggesting no family influences pre- or postnatally. Further, we found a dosage effect of time spent in the institution on health and developmental outcomes (Sand et al., 2024b; Lannen et al., 2026).

Furthermore, qualitative data indicated potential effects on their offspring: many reported difficulties when becoming a parent themselves, some reported that their children were taken away by authorities and placed in care, too. Many talked about wanting to make sure that their children do not suffer what they have gone through. Some decided not to have children at all as a result. This is in line with preliminary evidence that individuals formerly placed in institutions were much less likely to have children in the first place. Others talked about having managed to create stable, caring, loving homes for their children from that intention (Lannen et al., 2024).

#### 2.1.2 Assessment of offspring

##### 2.1.2.1 Eligibility and cohort description

Eligible are all offspring of formerly institutionalized individuals that participated in the assessment between 2019 and 2023, are still alive and have since not expressed either a wish to discontinue participation in the study or distress of some sort [*N* = 115/127 (90.5%)].

The 115 eligible individuals that were formerly institutionalized had between 0 and 4 offspring (M = 1.45, SD = 1.19). The total number of eligible offspring is *N* = 167. Offspring were born between 1974 and 2005 (M = 1988, SD = 5.75).

Preliminary power analyses yielded that for group comparisons (t-test for independent samples) with an alpha-error of 0.05 and a power of 0.80, at least 102 offspring are required to detect medium effect sizes. Regarding the main hypotheses, Fritz and MacKinnon (2007) state that medium effect sizes can be detected in a mediation model with *N* = 130.

For the quantitative data, the study works with the full sample. For the qualitative study part, it will invite a subset of the offspring to participate (*N* = 30), combining the following criteria to achieve a diverse sample: number of siblings, gender, growing up in various contexts (with parent(s) or out of home care, growing up in or outside Switzerland, being a parent themselves or not etc.). Comparable studies work with similar, or mostly even smaller numbers of interviews (Gabriel et al., 2021; Zöller et al., 2021).

##### 2.1.2.2 Contact procedure

In analogy to the study implemented with the formerly institutionalized individuals and parents of the current subjects, the recruitment process is set as an opt-out process, i.e., individuals will be contacted again for the next step unless the individual actively declines participation within a pre-determined timeframe.

###### 2.1.2.2.1 Consent from institutionalized individuals to contact offspring

The initial recruitment step involves a letter to the formerly institutionalized individuals with the aim to see whether they would agree for the research team to contact their offspring. There is information (flyer, website) available about the study for them to use to inform themselves and/or pass on to their offspring. If there is no response to the letter, the research team reaches out by telephone; if this is unsuccessful, they receive a final reminder letter offering an explicit opt-in option: they are asked to contact the study team. Otherwise, the participant is coded as passive decline. If they agree in principle, there is a standardized process for contacting their offspring.

###### 2.1.2.2.2 Contact with offspring

**Figure 2:**
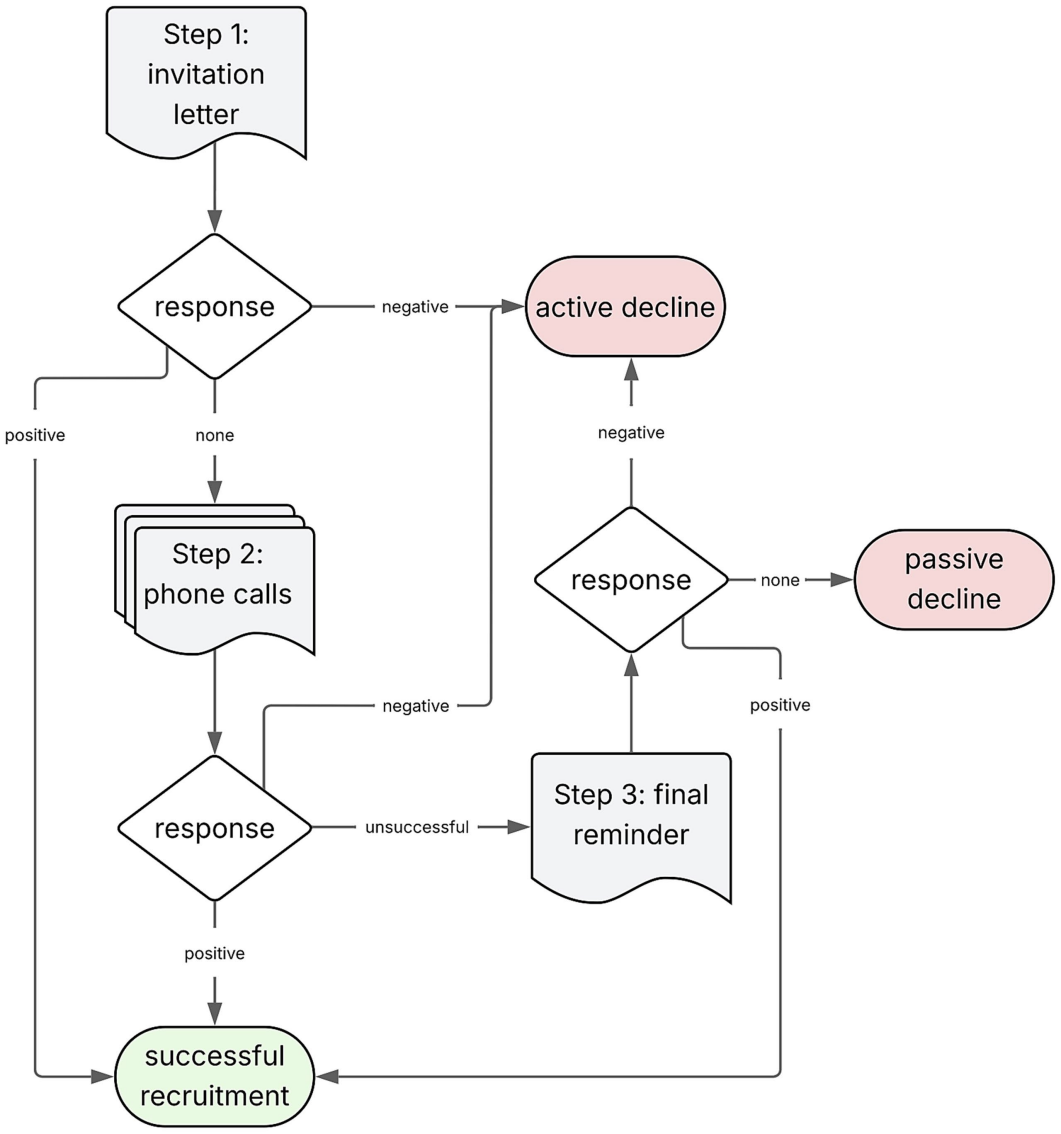
Recruitment process using a step-wise approach.

This process includes a step-wise approach with increasingly more information at each step to avoid overwhelm, in analogy to how this was done with the institutionalized individuals (see Figure 2) (Lannen et al., 2022).

Step 1: Invitation Letter: Offspring receive an initial letter with only minimal information about the study, announcing that they will receive a phone call with more information on the study within two weeks’ time, unless they let us know that they do not wish to be contacted again.

Step 2: Phone Calls: If offspring do not actively decline within two weeks, they receive a phone call. During the phone call, the researcher will go over the study details and answer any questions individuals may have. For details on how phone calls will be implemented see Lannen et al. (2022).

Step 3: Final Reminder: Based on feedback from individuals affected by institutional care collected as part of the initial preparatory phase (Lannen et al., 2020), rather than discontinue reaching out to them after five failed attempts to call them, one last letter is sent to offspring. The letter indicates that the study team has tried to reach them in vain and that, if they are still interested in participating in the study, they should get in touch with the study team (opt-in). The letter also includes a short version of the questionnaire. They are informed that they will not be contacted again unless they get in touch with the study team.

This has worked well for the study with the formerly institutionalized individuals, i.e., the parents of the current cohort: Out of 65 final reminder letters, 14 (21.5%) were glad to hear from the study team again and ended up participating in the study.

Step 4: Consent and data collection: Once an offspring agrees to participate, consent procedure and data collection are initiated.

##### 2.1.2.3 Research questions and hypotheses

###### 2.1.2.3.1 Quantitative data

The quantitative study arm focuses on the following research questions:

- Can intergenerational effects of early institutional placement be detected in offspring of institutionalized individuals in relation to their physical health, cognitive abilities, socioeconomic status and socioemotional health compared to offspring of non-institutionalized individuals?
- If so, what mediates and moderates these effects on the offspring?

**Figure 3:**
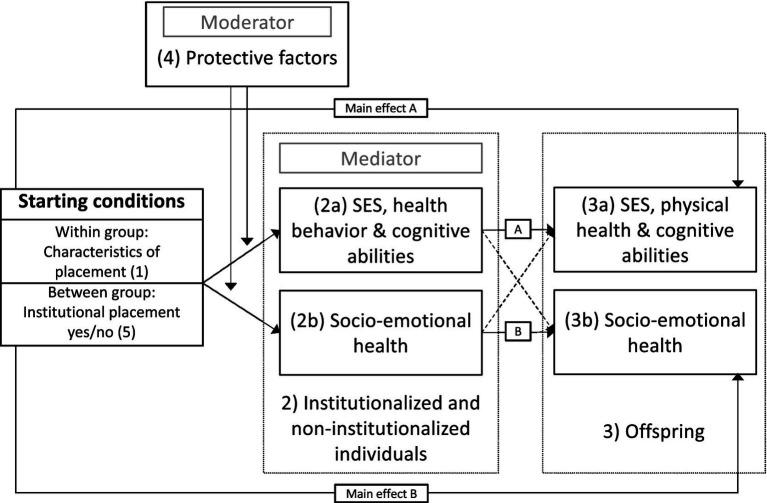
Conceptual model derived from the literature and preliminary analyses of cohort data.

Drawing from the literature outlined in the introduction as well as findings from the study with institutionalized individuals (section 2.1.1.2), a conceptual model was developed (Figure 3).

It is a moderated mediation model and makes assumptions for processes relevant within group (institutionalized cohort) and between groups (institutionalized cohort and non-institutionalized cohort).

The model assumes that outcomes of offspring are affected via outcomes by their parents (mediation part of the model).

The moderation part of the model is the switch that turns this chain reaction on and off. Hence, it can be assumed that if protective factors relevant for resiliency processes are active as moderators, the link between early care circumstances and offspring outcomes is no longer detectable. Early care circumstances *between group* (institutionalized individuals vs. non-institutionalized individuals) signifies whether an individual was placed in an institution or not. Early care circumstances *within group* (institutionalized individuals) are the specific characteristics of placements for an individual (such as duration of placement, interaction time or contact with family of origin).

Table 1 depicts operationalization of the variables in the model. Birth weight, age, gender and nationality will be entered as co-variates into the model.

**Table 1:** Construct and corresponding assessment instruments for the conceptual model depicted in Figure 1.

Conceptual model	Construct	Operationalization/instrument (Reference)
Care circumstances (1)	Placement Characteristics	Administrative data in Waves 1 & 2 *** Age at placement, duration of placement, quality of institutional care (as measured in interaction time per 24 h, child-staff ratio and size of the institution), reason for placement (single mothers and migrant worker status, poverty, invalidity) number of placement changes in different institutions up to age 14
Outcomes of institutionalized and non-institutionalized individuals (2a & 2b) and Offspring outcomes (3a & 3b)	Socio-economic status (SES)	Socio-demographic variables (education, income, civil status etc.) will be assessed in study-specific questionnaire based on the Swiss Household Panel (Voorpostel et al., 2016) and the Swiss Health Survey* (Bundesamt für Statistik, 2014)
	Physical health and health behavior	Physical health: 12-item Short Form Health Survey (SF-12)* (Morfeld et al., 2011) Anamnestic interviews** standardized physical exams** Health behavior: Study specific questionnaire on treatments, medication, health habits based on the Swiss Household Panel (Voorpostel et al., 2016) and the Swiss Health Survey* (Bundesamt für Statistik, 2014)
	Cognitive abilities	*Intelligence:* Wechsler Adult Intelligence Scale – Fourth Edition (WAIS-IV)** (Petermann and Petermann, 2013) *Fluency*: Materialien und Normwerte für die Neuropsychologische Diagnostik (MNND)** (Balzer et al., 2011) *Cognitive Flexibility*: Trail Making Test (TMT)** (Tombaugh, 2004) *Inhibition*: Stroop Test (ST)** (Strauss et al., 2006)
	Socioemotional health	*Mental health*: Brief Symptom Checklist (BSCL)* (Franke, 2017) *Relationships and attachment*: Adult Attachment Scale (AAS-R)* (Schmidt et al., 2016) Relationship questionnaire (RQ)* (Bodenmann et al., 2011) Parental Caregiving Style Questionnaire (PCS-Q)* (Neumann, 2002) *Emotion regulation*: Cognitive Emotional Regulation Questionnaire (CERQ)* (Loch et al., 2011)
Offspring only outcomes (3b)	Socioemotional health	*Adult attachment to parents*: Adult Scale of Parental Attachment (ASPA)* (Michael and Snow, 2019) *Parenting style*: Alabama Parenting Questionnaire – German Version (DEAPQ-EL-GS)* (Reichle and Franiek, 2009) *Adverse childhood experiences*: Childhood Trauma Questionnaire (CTQ)* (Wingenfeld et al., 2010b) Adverse Childhood Experiences (ACE)* (Wingenfeld et al., 2010a) *Childhood circumstances*: Questions regarding institutional placement, childcare, frequent moves and staying abroad*
Protective factors (4)		*Psychological protective factors*: Resilience Scale (RS)* (Schumacher et al., 2004) Sense of Coherence Scale (SOC-L9)* (Schumacher et al., 2000) Self-efficacy Scale (ASKU)* (Beierlein et al., 2012) *Individual protective factors*: Amount of contact with biological family during institutional stay (historical research records Wave 1 & 2)***
Institutional placement (5)	Placement yes/no	Assignment to either MMI cohort or ZLS cohort

*will be part of the online questionnaire battery, ** will be part of an in-person neuropsychological assessment, *** was retrieved from historical records.

Based on the conceptual model depicted in Figure 3, the following hypotheses can be formulated:

Hypotheses: between-group

1. Main effect A: Offspring of institutionalized individuals report worse physical health, cognitive abilities & SES than offspring of non-institutionalized individuals.
2. Mediation effect A: Differences regarding physical health, cognitive abilities and SES between offspring of institutionalized and non-institutionalized individuals can be explained by their parent’s SES, cognitive abilities and health behavior.
3. Main effect B: Offspring of institutionalized individuals report worse socioemotional health than offspring of non-institutionalized individuals.
4. Mediation effect B: Differences regarding socioemotional health between offspring of institutionalized and non-institutionalized individuals can be explained by their parents’ socioemotional health.
5. Differential effects are hypothesized the same as in the within group model (Differential effects 1 and 2).
  - Moderated mediation: All main and mediation effects are buffered by protective factors related to institutionalized and non-institutionalized individuals.

Hypotheses within-group:

1. Main effect A (direct effect): Early care circumstances affect physical health, cognitive abilities and SES of offspring.
2. Mediation effect A: SES, health behavior and cognitive abilities of institutionalized individuals mediate the main effect of early care circumstances on physical health, cognitive abilities and SES of offspring.
3. Main effect B: Early care circumstances affect socioemotional health of offspring.
4. Mediation effect B: Socioemotional health of institutionalized individuals mediates the main effect of care circumstances on socioemotional health of offspring.
  - Differential effect 1: Mediation effect A and Mediation effect B: Mediation effect B is stronger than mediation effect A due to the differential effects of institutional placement on the different developmental domains in early childhood.
  - Differential effect 2: Cognitive abilities, health behavior and SES of institutionalized individuals (2a) impact offspring’s socioemotional health (3b) and socioemotional health of institutionalized individuals (2b) impacts offspring’s physical health, cognitive abilities and SES (3a), however, these relationships are expected to be less strong than pathways between 2a and 3a & 2b and 3b.
5. Moderated mediation: All main and mediation effects of early care circumstances are buffered by institutionalized individuals’ protective factors such as a sense of self-efficacy and a sense of coherence as well as family contact during institutionalisation.

###### 2.1.2.3.2 Qualitative data

Placement: What do we learn from offspring’s narratives about the placement in infant care institutions of their parents?

Subjective impact of placement: Do they describe that it had an impact on their lives and if so, how? How do perspectives differ between parents and offspring? How do offspring describe their relationships with their parents?

Contextualizing with individuals growing up in families and zeitgeist: How did zeitgeist narratives around childhood and parenting shape the individuals’ experiences? What do we learn from comparing these findings with the experiences of parents, siblings and offspring of non-institutionalized individuals?

##### 2.1.2.4 Data collection

The consent and data collection process for offspring takes place between 2024 and 2026 and is closely aligned with what was done with the formerly institutionalized individuals which was published in the respective study protocol (Lannen et al., 2021). It includes a quantitative study arm with questionnaire and neuropsychological assessments and a qualitative study arm with interviews. For the in-person data assessment (neuropsychological assessments and interviews), data collection generally takes place at the MMI (implementing organization). Data collection takes place in German, Italian (or English and French, if necessary).

###### 2.1.2.4.1 Quantitative data

For the quantitative component, the study will work with the full sample and use questionnaire data as well as neuropsychological assessments, very similar to the assessment battery used for institutionalized individuals between 2019 and 2023.

In a first step, participants receive a comprehensive consent form and a link to fill in an online questionnaire. Questionnaires include information on demographics, physical and socioemotional health as well as protective factors (Table 1). In a second step, individuals are invited to participate in the neuropsychological assessment. Again, instruments are aligned with those collected for the formerly institutionalized individuals and will focus on neurocognitive outcomes (Table 1).

###### 2.1.2.4.2 Qualitative data

A subsample of the offspring participating in the quantitative study part will be invited to take part in an interview (for selection criteria see 2.1.2.1).

Interviews are conducted as semi-narrative interviews. These types of interviews are usually referred to as “semi-structured interviews” in English, but as we wish to emphasize the narrative element of the interview, we will use the term “semi-narrative” as a direct translation from the German „teilnarrative Interviews “(Helfferich, 2011). In these interviews, we will ask open-ended and broad questions according to Rosenthal (Rosenthal, 2015) and related to pre-defined themes. For example, a question could be: “What do you know about the institutional placement of your mother/father?.” The method of semi-narrative interviews therefore allows the interviewee a maximum degree of freedom in terms of content and course of narrations within a defined framework of questions.

This approach was chosen to provide participants with an open narrative space in which they can recount their life stories in their own words and according to their own priorities, without imposing a predefined theoretical or thematic structure. The aim is not to reconstruct an objective or complete account of past events, but to understand how offspring narrate, interpret, and integrate their early institutional placement into their life stories from their present perspective.

Rather than treating these elements solely as sources of bias, the biographical-narrative approach considers them part of the phenomenon under investigation: they show how early institutionalisation is remembered, reconstructed, silenced, transmitted, or given meaning over the life course.

Ample time will be made available so that the interviewee can talk about their personal experiences in detail (Kruse, 2011).

The length of the interview is determined by the interviewee and may last up to several hours. The interviewees decide what they do and do not want to share. Themes that will be covered in the interview questions include:

- Narrative and contextualization of institutional placement in the family (if at all)
- Attitudes toward and relationship with their parents (formerly institutionalized individuals)
- Thoughts about parenthood in general (before becoming a parent, if at all)
- Additional relevant themes (schooling and educational trajectory, friendships, relationships, critical life events)
- Taking stock of one’s life so far

Where several offspring from the same family participate, the qualitative analysis will also attend to intra-family differences in narratives, perceived family roles, parent–child relationships, birth-order-related experiences, and exposure to family stressors or protective factors over time.

##### 2.1.2.5 Comparison with Zurich Longitudinal Studies

Data for the comparison cohort not affected by institutionalization are collected as part of the Zurich Longitudinal Studies (ZLS) at the Child Development Center of the University Children’s Hospital in Zurich. In brief, the ZLS are a set of three comprehensive cohort studies on child growth, health, and development that are currently expanding into adulthood (Wehrle et al., 2021). They are considered one of the most comprehensive data sets on child development globally (Ulijaszek et al., 1998). Between 1954 and 1961, 432 healthy infants were enrolled in the first ZLS cohort (ZLS-1). Their physical, motor, cognitive, and social development and their environment were assessed comprehensively across childhood, adolescence, and into young adulthood. Altogether, *N* = 399 individuals from ZLS-1 are included as the comparison group to the formerly institutionalized individuals, excluding those who themselves were institutionalized as children (*N* = 10) or withdrew consent as adults (*N* = 23). In the 1970s, two further cohorts were added, with ZLS3 (second generation) being the cohort of offspring of ZLS 1 (Wehrle et al., 2021).

Since 2019, the participants of the ZLS cohorts have been traced and invited to participate in an assessment in adulthood to investigate their current health and development. Data for ZLS-1 on health and development was collected from *N* = 202 individuals (response rate 60%) between 2019 and 2021, parallel to the effort of the data collection with formerly institutionalized individuals. After excluding participants with institutionalisation experiences during childhood, *N* = 197 individuals remain for comparison with the formerly institutionalized group. Particularly relevant for this project is the ZLS-3 (“Generational Study”), a cohort born between 1973 and 2002 and including 296 offspring of the ZLS-1 participants. Thus, the ZLS-3 will serve as the comparison group for the offspring of those placed in institutions, while the ZLS-1 as the comparison for institutionalized MMI individuals. Data collection for the 289 eligible members of the ZLS-3 cohort also takes place between 2025 and 2027 and 286 of them (offspring of all ZLS-1 participants that were never institutionalized during childhood) serve as a comparison group to the offspring of the formerly institutionalized individuals. Notably, instruments used have been aligned between the two cohorts of institutionalized and non-institutionalized individuals from the onset of the studies in the 1950s, including for offspring. To ensure comparability between cohorts and minimize selection bias, offspring of ZLS-1 will be matched with those from the formerly institutionalized cohort based on parental age and sex.

### 2.2 Data analysis

#### 2.2.1 Data from questionnaires and neuropsychological assessments

Quantitative data from questionnaires and neuropsychological assessments will be analysed using state of the art longitudinal statistical techniques such as latent growth models, latent mediation models, latent moderation models, or random-intercept cross-lagged models (Hamaker et al., 2015). Missing data will be addressed using full-information maximum likelihood or multiple imputation, depending on the pattern of missingness at hand (Graham, 2009). The clustered nature of the data will be taken into account by applying a correction to the standard errors of the respective models (Freedman, 2006).

Given the quasi-experimental design, all quantitative analyses will be interpreted with caution regarding causal inference. The primary analyses will examine associations between parental infant institutionalisation, proposed mediators and moderators, and offspring outcomes. Where available, variables reflecting later life trajectories and broader family context, including socioeconomic conditions, health behavior, socioemotional health, relationship and attachment-related factors, adverse childhood experiences, childhood circumstances, family life events, and protective factors such as social support, will be included as covariates, mediators, moderators, or contextual variables, depending on the specific model. Sensitivity analyses will be conducted where sample size and data structure allow, to examine whether associations remain robust when these factors are considered.

Given the systemic nature of intergenerational transmission, associations between the formerly institutionalised parent and offspring outcomes will not be interpreted as parent-specific effects in isolation. In addition, analyses will explicitly consider the gender of the formerly institutionalised parent. Parent gender will be included as a covariate in the main models and, where sample size permits, examined as a potential moderator of mediation pathways. This will allow us to explore whether associations between parental institutional placement, proposed mediators, and offspring outcomes differ for maternal versus paternal institutionalisation. Because information on the other parent and coparenting dynamics is limited in this historical cohort design, and because gender-specific subgroup sizes may be small, these analyses will be considered exploratory and interpreted cautiously.

In addition to between-group comparisons, intra-family variability will be explored where the data allow. For families with more than one participating offspring, we will descriptively and exploratorily examine whether offspring outcomes vary within families according to factors such as offspring gender, birth order, age spacing, parent–child relationship characteristics, or periods of differing family circumstances. Given the limited number of sibling clusters, these analyses will not be used as confirmatory tests, but as complementary analyses to contextualize heterogeneity in developmental trajectories and potential mechanisms of vulnerability and protection.

Potential recruitment-related selection bias will be examined descriptively by documenting recruitment flow, including parental consent to contact offspring, passive and active refusal, non-response, and offspring participation. Where possible, available characteristics of formerly institutionalised parents who do and do not consent to offspring contact, as well as participating and non-participating offspring, will be compared. These analyses will be used to assess the possible direction of selection bias and to inform the interpretation of findings. Overall, mediation and moderation analyses will be understood as exploratory and hypothesis-guided rather than as definitive tests of causal, parent-specific, or gender-specific transmission.

#### 2.2.2 Interview data

The semi-narrative interviews will be transcribed verbatim using the simple transcription conventions by Dresing and Pehl (Dresing and Pehl, 2018). If required, specific interviews or passages will be additionally transcribed according to Bohnsack et al. (2013) for in-depth fine analyses. The analysis will be guided by the principles of interpretative social research. The aim of this approach and its reconstructive analysis procedure is to understand the world from the perspective of the acting persons and to work out how people construct their reality (Rosenthal, 2015). Guided by this approach, the transcripts will be analysed using a sequential abductive procedure (Rosenthal, 2015). We will explicitly consider the retrospective and narrative character of the data. Particular attention will be paid to narrative structure, temporal positioning, emotional tone, omissions, contradictions, changes in perspective, and references to family stories or collectively shared narratives. Where possible, we will distinguish between personally remembered experiences, accounts transmitted by others, information derived from records, and retrospective interpretations. After each interview, interviewers will write memos documenting the interview context, interactional dynamics, and possible indications of social desirability, suggestion, or other contextual influences. These memos will be used as part of the interpretive process.

This approach allows for a valid analysis in qualitative research even with a small number of interviews conducted for each type of data source. The approach of the reconstructive and abductive analysis is based on the principle of openness and the goal is to discover new combinations of features for which no existing body of knowledge, explanations, or rules can yet be found (Kruse, 2011). Hypotheses and theories will be generated abductively as part of the analysis process and will be continuously tested throughout the analysis process (Rosenthal, 2015), rather than formulated *a priori* and then tested as is customary for quantitative research. However, there are overarching research questions and themes that will be answered (see section 2.1.2.4). In addition, content analyses (Mayring and Fenzl, 2019) will be conducted in order to process larger amounts of data and in order to stay close to the expressions of the individuals themselves which lends itself to using a deductive approach (Kilic and Lannen, in revision).

#### 2.2.3 Data triangulation

The main objective of the triangulation will be to see how key findings in the quantitative analyses are reflected in the qualitative interviews, i.e., are findings confirmed in the subjective view of the participants or can contradictory statements be found and vice versa. We will also remain open for new or more differentiated insights from the interviews that might not be detected in the quantitative data. Qualitative data can also be referred to when quantitative results are seemingly counter-intuitive or else to provide illustrative example for quantitative results. In addition, we will be able to triangulate qualitative data from different sources within a family system and trace different perspectives on the situation by putting statements in relation to each other along the research questions. Lastly, stories from offspring affected by institutionalisation and stories of offspring not affected by institutionalisation will be put in relation to each other.

## 3 Discussion

### 3.1 Scientific relevance

This study presents a unique opportunity to investigate the intergenerational outcomes of early institutional placement under psychosocial deprivation, building on a 60-year longitudinal assessment of individuals placed in infant care institutions in the 1950s in Switzerland. It extends the inquiry beyond the directly affected individuals to examine how such early experiences may be transmitted into the next generation. To our knowledge, no comparable dataset or study exists globally that combines this depth of follow-up, methodological rigor, and intergenerational reach.

The study addresses several limitations common to prior research.

Most notably, it will differentiate our understanding of how a highly specific event, namely institutional placement under psychosocial deprivation, affects individuals and their offspring over time.

The study design also enables the exploration of hypothesized intergenerational pathways of transmission, as illustrated in Figure 3. Rather than assuming a single pathway, we distinguish between several possible mechanisms. Early institutional placement may be associated with socioeconomic conditions, health behavior, physical health, and cognitive functioning in the formerly institutionalised generation, which may subsequently be related to socioeconomic, health-related, and cognitive outcomes in their offspring. In parallel, early psychosocial deprivation may be linked to socioemotional development, including emotional regulation, attachment-related functioning, and interpersonal relationships, which may influence the emotional and relational context of the next generation. In addition, concepts from social learning theory, internalized relational schemas, parental representations, and expectancy processes may help explain how early experiences of being cared for — or of not being responded to — shape later caregiving behavior, parent–child interactions, and expectations of children’s needs. Protective factors, such as supportive relationships, educational opportunities, or later stable caregiving environments, will be examined as potential moderators that may buffer, weaken, or interrupt these pathways. Mediation and moderation analyses will therefore be used exploratively to examine possible mechanisms of intergenerational transmission and to identify conditions under which risk and resilience unfold across generations.

Considering that transmission of events across generation takes place, not least as a result of interaction patterns, placement in infant care institution under deprivation is particularly relevant in this regard, as psychosocial deprivation affects the very patterns of interaction in early life that are later on relevant when having children of one’s own. Also, this design will allow us to look at how events affect the next generation individuals past their own childhood into early adulthood.

Another strength lies in the parallel community sample cohort of individuals who grew up in their families of origin. Their inclusion enables differentiation between the effects of institutionalisation and those attributable to broader historical or social factors. This natural control group significantly enhances the study’s capacity to isolate the unique contributions of early institutional psychosocial deprivation.

The use of a mixed-methods design by combining quantitative and qualitative data enables the generation of new hypotheses, deepen quantitative results and reconstruct the stories of those affected.

Most notably, it avoids self-selection bias by drawing from a population-based sample whose long-term follow-up has shown no signs of selectivity. Unlike studies that recruit participants via calls for reparation or public appeals—methods that may introduce biases related to awareness, motivation, or emotional salience—this study benefits from a systematic, archival recruitment strategy implemented through population registry.

### 3.2 Anticipated challenges

Although preliminary inquiries showed satisfactory interest in study participation, possible sample attrition remains a key risk to the study. This is accentuated by the fact that offspring are recruited through their formerly institutionalised parents. This recruitment strategy may introduce selection bias, as it partly depends on whether parents are willing and able to allow contact with their offspring and whether offspring are reachable and willing to participate. Families characterized by conflict, estrangement, broken ties, or particularly vulnerable life trajectories may therefore be underrepresented. As a result, the sample may overrepresent families with relatively more intact parent–offspring relationships, introducing a potential optimism bias and possibly leading to an underestimation of intergenerational difficulties.

This bias has important implications for interpretation. If differences between offspring of formerly institutionalised and non-institutionalised individuals are observed despite this potential selection process, the true extent of intergenerational difficulties may be underestimated. Conversely, if no or only weak differences are detected, selective recruitment of more connected or functional families will need to be considered as a possible explanation. At the same time, the quality of the parent–offspring relationship is not merely a methodological concern, but may itself be part of the phenomenon under study. Difficulties in becoming a parent, maintaining relationships with offspring, or sharing family history may represent potential long-term consequences of early psychosocial deprivation.

A related limitation concerns causal inference. Although the study includes a comparison group and builds on a well-documented quasi-experimental historical cohort, offspring outcomes cannot be attributed directly to parental infant institutionalisation. Cognitive, social, emotional, and health-related outcomes in offspring are likely to result from complex interactions between multiple individual, familial, and social determinants. These include the later life trajectories of formerly institutionalised individuals, family life events such as separations, bereavements or geographic mobility, socioeconomic conditions, relationship quality, social support networks, and characteristics of the other parent and the broader family system. Some of these factors may act as mediators or moderators, whereas others may constitute confounders or alternative explanatory pathways. Although the study will assess and integrate several of these dimensions where possible, residual confounding cannot be ruled out. Findings will therefore be interpreted cautiously as associations and potential pathways consistent with intergenerational transmission, rather than as evidence of direct causal effects.

To assess and mitigate these limitations where possible, recruitment flow will be documented in detail, including parental consent to contact offspring, passive and active refusal, non-response, and offspring participation. Where data are available, characteristics of formerly institutionalised parents who do and do not allow contact with offspring, as well as participating and non-participating offspring, will be compared. In addition, qualitative interviews will be used to contextualize family relationships, including narratives of closeness, conflict, silence, estrangement, and the transmission or non-transmission of family history.

A further, related risk concerns the continued stigma surrounding early institutionalisation and compulsory social measures. This stigma may influence whether formerly institutionalised individuals have shared their history with their offspring and may also affect their willingness to allow researchers to contact them. At the same time, such silence, partial disclosure, or non-transmission of family history will be treated as analytically relevant in the qualitative component of the study.

Due to the fact that about half of the original cohort of institutionalized individuals were children of migrant workers, a substantial number of offspring are expected to live abroad in their parents’ country of origin. This will unavoidably bring some challenges for data collection in terms of travel, availability of instruments in multiple languages and language skills of the researchers.

However, the use of a well-documented, population-based sample, together with rigorous analytic strategies and the highly trained staff proficient in multiple languages enhances the reliability of the findings. Ethical considerations—especially in working with family members and retrospective data—are also paramount, and the study protocol includes detailed procedures for informed consent, data protection, and participant wellbeing that has, in the cohort of institutionalized individuals, i.e., parents of the current cohort, successfully enabled mitigating ethical risks.

### 3.3 Broader impact

In addition to its scientific contributions, the study bears significant historical and societal relevance. It contributes to the process of reconciliation and rehabilitation related to Compulsory Social Measures in Switzerland before 1981. By leveraging access to historical records and longitudinal data, the project provides a data-driven approach to understanding not only the consequences of these practices but also their socio-cultural origins. In doing so, it supports societal reflection and may sensitize practitioners and policymakers to potential blind spots in current care systems. It is one of many international efforts for reconstruction and reconciliation of state measures related to institutional placements (Wright et al., 2025). This study uniquely highlights not only the consequences of these measures to those placed in institutions, but how these practices may have affected the broader family system and the next generation offspring.

Today, millions of children worldwide continue to grow up in residential care institutions (Desmond et al., 2020). Although care conditions in Western countries have changed substantially, the findings of the present study remain relevant, as many children in low- and middle-income countries still live in institutional settings that resemble the conditions examined in this project (Lee, 2000; The St. Petersburg–USA Orphanage Research Team, 2005; Berument, 2013; Koch and Franzsen, 2017).

The findings of this study—on long-term consequences, mediating mechanisms, and protective processes—will inform practice and policy locally as well as internationally. They will provide a deeper understanding of how to support children and families affected by institutional care, how to mitigate long-term harm, and how to foster resilience in those growing up under adverse circumstances.

Most importantly, the study will allow individuals affected by institutional placement practices and their offspring to have a voice, or as Becker-Blease and Freyd (2006) put it (p. 225): “To the extent that silence is part of the problem - silence impedes scientific discovery, helps abusers and hurts victims - then this is no trivial matter.” The high relevance of investigating the past lies in the recognition of what has happened as unjust. In this way, the right of those affected for recognition of past suffering receives central relevance in relation to the society’s upholding of basic human rights (Ziegler et al., 2018, p. 12). Or to quote Wensierski (2006, p. 6): After many decades, it appears to be of utmost importance to the victims to finally speak freely, and in that way to free themselves of the framework of oppression.“.

## 4 Dissemination

The results bear significant potential for scientific, practical, and social impact at national and international levels, and results will be disseminated accordingly.

To this end, scientific publications focused on the key research questions will be published in peer-reviewed journals related to medicine, psychology and educational and other social sciences, and results will be presented at national and international scientific conferences of these disciplines. In addition, scientific findings and evidence-based solutions to various challenges that can arise during the practical implementation of research projects will be published as well.

Furthermore, a significant effort will be made to make results accessible to a nonscientific audience including professionals working with children, government agencies and NGOs that are nationally and internationally active on child protection issues and institutional placements of children today. It will also target organizations concerned with reconciliation processes of historical state measures and institutional placements internationally as well as survivors of such measures.

Evidence Briefs will serve as a key tool for disseminating results to a nonscientific audience. In addition, a book will be published that narrates the life stories of affected individuals across generations. Efforts will be made to develop a curriculum for professionals working with children (psychology, social work, educational sciences) and with children in primary and secondary school settings.

## Data Availability

The original contributions presented in the study are included in the article/supplementary material, further inquiries can be directed to the corresponding author.
